# Developing an integrated approach based on geographic object-based image analysis and convolutional neural network for volcanic and glacial landforms mapping

**DOI:** 10.1038/s41598-022-26026-z

**Published:** 2022-12-10

**Authors:** Mohammad Kazemi Garajeh, Zhenlong Li, Saber Hasanlu, Saeid Zare Naghadehi, Vahid Hossein Haghi

**Affiliations:** 1 Department of Remote Sensing and GIS, University of Tabriz, Tabriz, Iran; 2 Geoinformation and Big Data Research Laboratory, Department of Geography, University of South Carolina, Columbia, SC USA; 3 Department of Civil, Environmental and Geomatics Engineering, College of Engineering and Computer Science, Florida Atlantic University, 777 Glades Road, Boca Raton, FL 33431 USA; 4 Department of Geography and Urban Planning, University of Tabriz, Tabriz, Iran

**Keywords:** Environmental sciences, Planetary science, Solid Earth sciences

## Abstract

Rapid detection and mapping of landforms are crucially important to improve our understanding of past and presently active processes across the earth, especially, in complex and dynamic volcanoes. Traditional landform modeling approaches are labor-intensive and time-consuming. In recent years, landform mapping has increasingly been digitized. This study conducted an in-depth analysis of convolutional neural networks (CNN) in combination with geographic object-based image analysis (GEOBIA), for mapping volcanic and glacial landforms. Sentinel-2 image, as well as predisposing variables (DEM and its derivatives, e.g., slope, aspect, curvature and flow accumulation), were segmented using a multi-resolution segmentation algorithm, and relevant features were selected to define segmentation scales for each landform category. A set of object-based features was developed based on spectral (e.g., brightness), geometrical (e.g., shape index), and textural (grey level co-occurrence matrix) information. The landform modelling networks were then trained and tested based on labelled objects generated using GEOBIA and ground control points. Our results show that an integrated approach of GEOBIA and CNN achieved an ACC of 0.9685, 0.9780, 0.9614, 0.9767, 0.9675, 0.9718, 0.9600, and 0.9778 for dacite lava, caldera, andesite lava, volcanic cone, volcanic tuff, glacial circus, glacial valley, and suspended valley, respectively. The quantitative evaluation shows the highest performance (Accuracy > 0.9600 and cross-validation accuracy > 0.9400) for volcanic and glacial landforms and; therefore, is recommended for regional and large-scale landform mapping. Our results and the provided automatic workflow emphasize the potential of integrated GEOBIA and CNN for fast and efficient landform mapping as a first step in the earth’s surface management.

## Introduction

Landforms are the result of interior and exterior forces of ecological, hydrological, geomorphological, and geological processes that shape the Earth’s surface^1^. The shapes and sizes of volcanic landforms range from small scoria cones to large flood basalt^2^, which are classified into polygenetic and monogenetic volcanoes^3^. The first type includes a shield, composite, and caldera volcanoes. The second category includes tuff rings and cones, mafic tiny centres (e.g., scoria and spatter cones), and maars and kimberlites^4^. Volcanoes are among the most complicated and dynamic landforms. Tectonic setting, eruption way, lava composition and volume, surface environment, and age contribute to the significant morphological diversity of volcanic landforms^5^. Volcanoes affect the terrain in the surrounding areas. Volcanism influences the landscape of a region in many ways, including the patterns and types of fissures and vents, the duration of its activity, the relative age of the volcanism, the composition and physical characteristics of the extruded material, and the amount and extent of erosion^6^. Therefore, their locations and features must be detected and mapped.

Glacial landforms in mountains are a critical water source for the future, particularly in semi-arid and arid regions^7^. Studies have revealed that approximately 2.15% of the world's drinkable water is stored as ice in polar and mountainous glaciers, with their residence times ranging from 20 to 100 years^8,9^. Typically, glacial landforms can be determined using a combination of the visible and shortwave infrared band ratios and Synthetic Aperture Radar (SAR) and topographic datasets^10^. These techniques, on the other hand, are insufficient to detect and map glacial landforms, which are spectrally indistinguishable from the surrounding paraglacial terrain^11^. In addition, atmospheric and earth factors may affect the spectral reflectance of glacial landforms^12^. Therefore, accurate information and inventories of glacial landforms are essential to their management.

Advances in satellite remote sensing methods have provided accurate information from vast and inaccessible volcanic areas. Satellite remote sensing overcomes the limitations of field-based methods such as global positioning system (GPS), which can only be applied locally and during specific seasons and months. Using this method, volcanoes can also be monitored and mapped despite their remoteness and harsh nature^13,14^. In previous studies, machine learning methods^15–19^ and object-based image analysis (OBIA)^6,13,14,20,21^ were used to detect and map the Earth’s landforms. Image classification techniques based on machine learning have provided accurate information about the Earth’s features^22^. As a strong alternative to traditional statistical methods, machine learning techniques that combine computational power with big data, are able to capture non-linear behaviors and learn as new data arrive^23^. These techniques like Support Vector Machine (SVM) and logistic regression, on the other hand, need to pre-process the datasets using Histogram of Oriented Gradients methods or smoothing filters to overcome a specific classification problem. Additionally, the detection of the Earth’s features is largely carried out at the pixel level in machine learning-based approaches^24^. Since landforms are representative of complicated features, expert knowledge plays a key role in the accuracy of their detection with conventional rule-based methods in geographic object-based image analysis (GEOBIA)^25^. Nonetheless, determining the appropriate thresholds for grouping objects into landforms based on each geomorphological diagnostic factor is a challenging task^26^. Classification methods using only spectral information, such as the parallelepiped, the maximum likelihood, and the minimum distance from the mean are not sufficient for analyzing multispectral images at high resolutions. An object with a given earth’s feature tends to be represented by pixels with heterogeneous spectral reflectance characteristics in high spatial resolution images^27^.

Recently, various machine learning-driven methods such as deep learning have been integrated with GEOBIA for the detection and mapping of land use/cover^28–31^, gully erosion^32,33^, and landslide^34–36^. According to the literature review, there is a limited number of research explored the efficiencies of an integrated GEOBIA and convolutional neural network (CNN) to delineate volcanic and glacial landforms.

## Aim

In this study, we investigate the performance of an integrated GEOBIA and CNN approach for volcanic and glacial landforms mapping in Sahand Volcano, Iran by using Sentinel-2 imagery as the foundational dataset and secondary data, such as the Digital Elevation Model (DEM), slope, aspect, flow accumulation, and curvature. We also intend to compare the effectiveness of Ground Control Points (GCPs) gathered from the study area and objects that were generated by GEOBIA for training and validating the landform’s CNN model.

## Datasets and methodology

### Datasets

**Figure 1:**
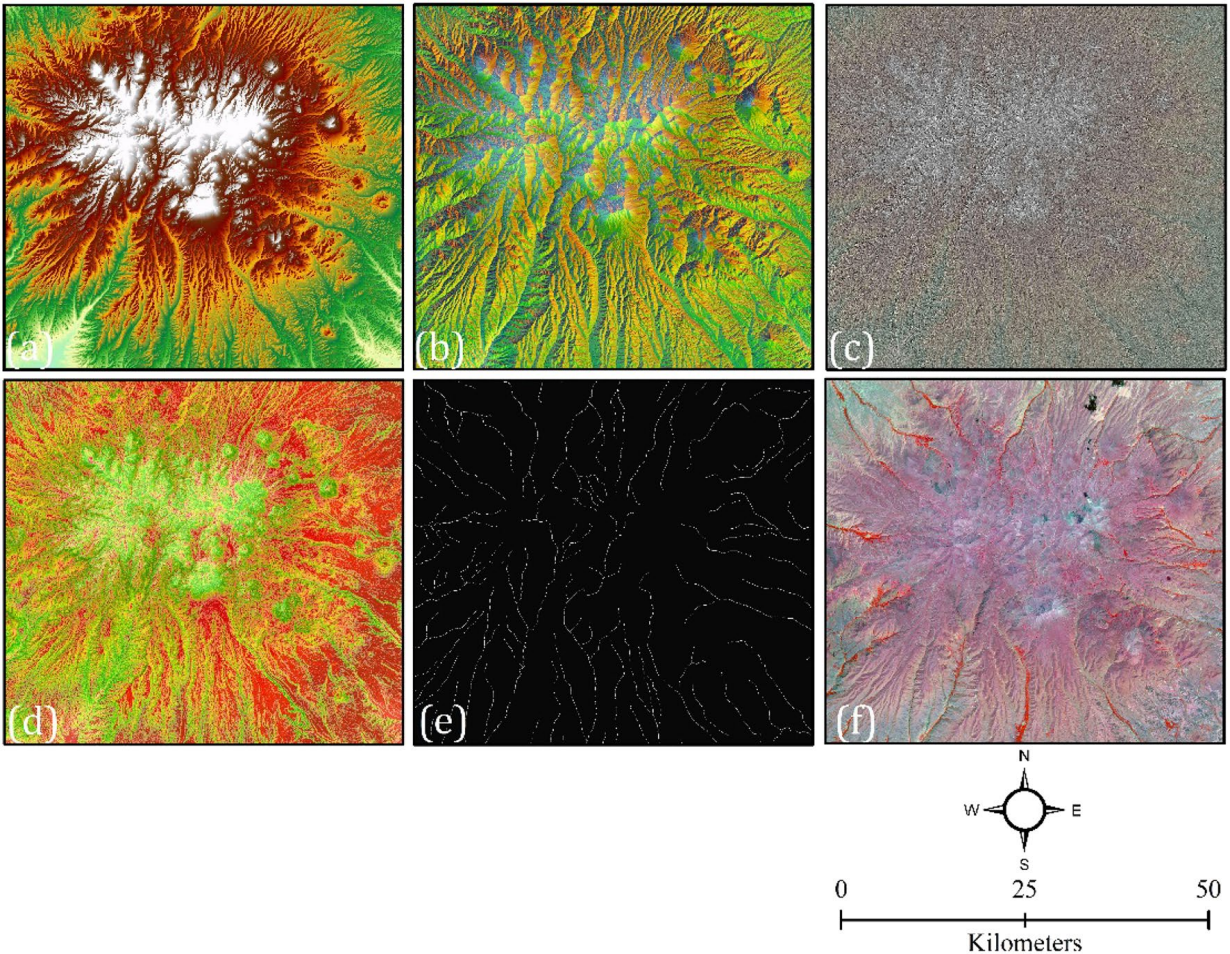
Various predisposing variables for volcanic and glacial landforms mapping, including (**a**) DEM, (**b**) aspect, (**c**) curvature, (**d**) slope, (**e**) flow accumulation, and (**f**) Sentinel-2 image.

For volcanic and glacial landforms mapping, we acquired freely available Sentinel-2 imagery (with bands 2 (Blue), 3 (Green), 4 (Red), and 8 (NIR)). Although a high-resolution, freely available DEM for the study area exists (with spatial resolution of 12.5 m), it was necessary to have consistent and comparable datasets that would be applicable to other locations. Due to this, a national topographic map at a scale of 1:25,000 was used to drive DEM. With the spatial analysis carried out in the ArcGIS environment, secondary datasets were generated, including aspect, slope, curvature, and flow accumulation with a spatial resolution of 12.5 m (Fig. 1). In Table 1, we list the characteristics of predisposing variables for volcanic and glacial landforms.

**Table 1:** Characteristics of predisposing variables for volcanic and glacial landforms mapping.

Characteristics	Properties	Source
Sentinel 2	Bands 2, 3, 4 and 8 (10 m)	www.glovis.com
DEM	12.5 m	Topographic map
Aspect	12.5 m	DEM
Curvature	12.5 m	DEM
Slope	12.5 m	DEM
Flow accumulation	12.5 m	DEM

To train the CNN models, we used an inventory map of volcanic and glacial landforms, delineated outlines generated from semi-automated GEOBIA, and ground control points (GCPs). All 935 GCPs (Fig. 7) were collected from the study area with GPS, geomorphological maps, and Google Earth to validate the accuracy of GEOBIA-generated objects and CNN. 70% of these datasets were used to train models, while the remainder (30%) were employed to verify classification accuracy.

### Methodology

**Figure 2:**
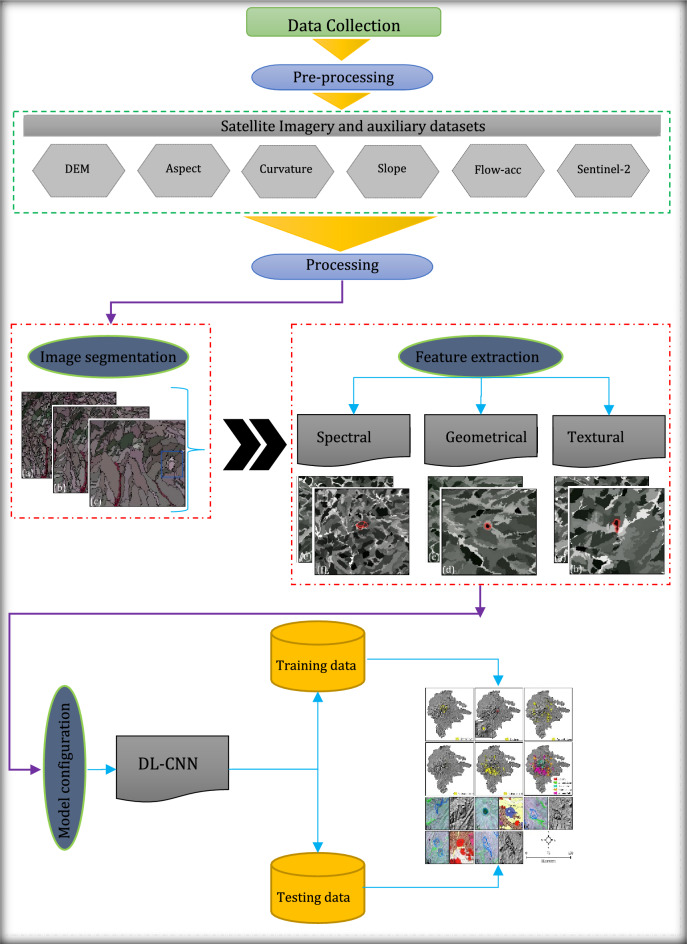
An overview of the methodology used for detecting and mapping volcanic and glacial landforms.

An overview of the methodology for detecting and mapping volcanic and glacial landforms is shown in Fig. 2. In the first step, we segmented our datasets using the Multi-Resolution Segmentation (MRS) algorithm in the eCognition software (www. geospatial.trimble.com). Our next step was to generate landform image objects based on geometrical, textural, spectral and contextual features in GEOBIA. To train the landform CNN models, not only generated objects from GEOBIA but also an inventory map of volcanic and glacial landforms as well as GCPs were used. Finally, seven evaluation indexes, including intersection over union (IOU) values, recall (RC), precision (PC), specificity (SP), F-measure (FM), accuracy (ACC), kappa (KP), Fivefold cross validation as well as fuzzy synthetic evaluation (FSE) were employed to validate the accuracy of the classification results.

### Geographic object-based image analysis

GEOBIA is an image analysis paradigm, which relies on groups of homogeneous pixels to classify images’ features^37^. These image objects reveal real-world entities that are tested based on their textural, spectral, contextual, and geometrical features. Image objects are then classified on the basis of their features using a cohesive methodological platform, integrating multi-scale regionalization methods augmented with nested representations and rule-based classifiers^38^. Such an integrated framework is linking digital-based remote sensing and vector-based GIS domain^39^. GEOBIA includes two main steps in this study, image segmentation and feature extraction.

### Image segmentation

**Figure 3:**
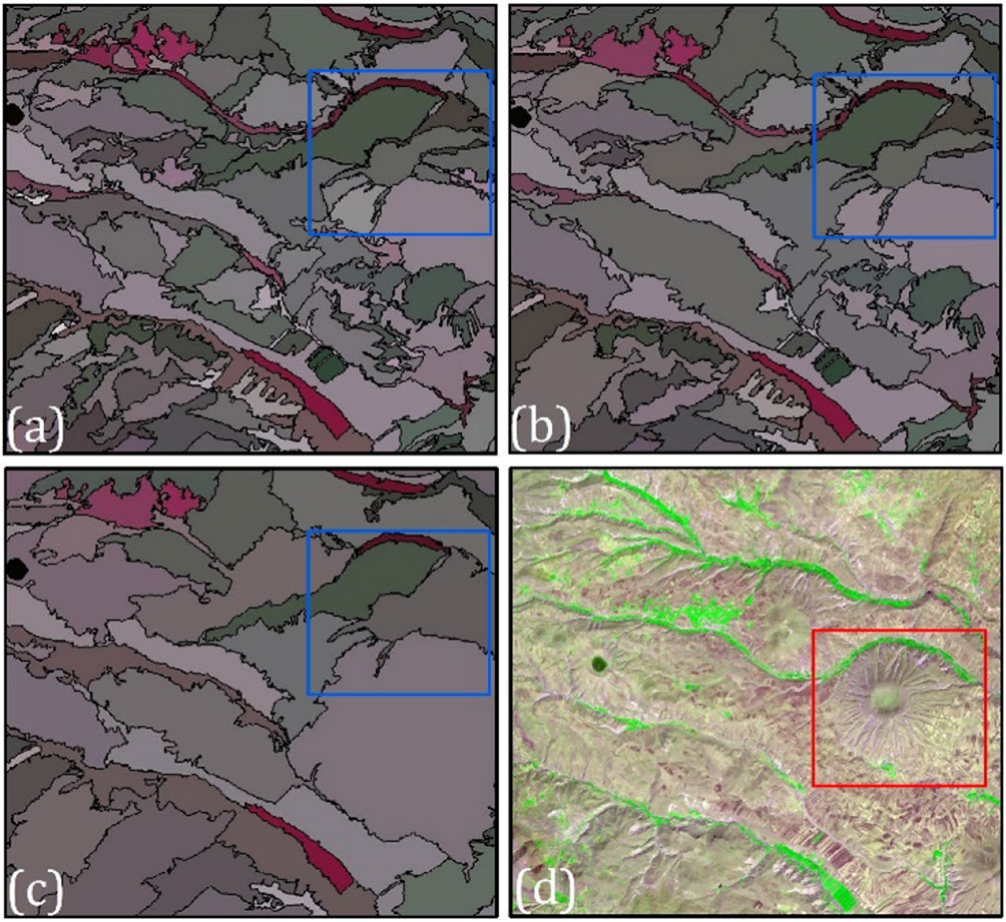
Segmentation results for, (**a**) ENVI feature extraction, (**b**) eCognition ESP2, (**c**) GRASS GIS SPUS PO, and (**d**) original Sentinel-2 image.

It has been argued that determining an optimal segmentation parameter value is a heuristic, subjective, challenging, and time-consuming process in the GEOBIA literature^40–42^. As a result, several GEOBIA software options have been developed to increase objectivity and automate the process of determining the optimal value, which are categorized into two groups namely free and open-source (GRASS GIS, Orfeo Toolbox, InterIMAGE, and RSGISLib) and commercial software (Trimble eCognition, L3Harris Geospatial ENVI Features Extraction, Esri ArcGIS Pro, and PCI Geomatica) options^43^. Based on an overview of over 200 GEOBIA-based researches for earth’s features classification^40^, found Trimble eCognition software the most popular (81%) image-segmentation method for satellite-based classification, while 4% employed L3Harris Geospatial ENVI Features Extraction. Therefore, this study used eCognition software to extract landform-derived objects for CNN training. Table 2 represents a brief description of all available software/tools for object-based segmentation. Figure 3 also portrays segmentation results for eCognition ESP2, ENVI Feature Extraction, and GRASS GIS SPUS PO.

**Table 2:** All available software/tools, their algorithms, and source for object-based segmentation.

Software/tool	Algorithm	Availability	Developer/Reference
InterSeg	Region-based	Available upon request	Happ et al. (2016)
SEGEN	Region-based	Commercial	Gofman (2006)
BerkeleyImgSeg	Region-based	Commercial	Clinton et al. (2010)
Orfeo Toolbox	Region-based	Freeware	Grizonnet et al. (2017)
RHSeg	Region-based	Evaluation copy	Tilton et al. (2012)
IMAGINE Spatial Modeller	Edge-based	Commercial	Hexagon geospatial
ENVI Feature Extraction	Edge-based	Commercial	Harris Geospatial Solutions
IDRISI GIS Tool	Edge-based	Commercial	Clark Labs
GRASS GIS	Region and edge-based	Freeware	Neteler et al. (2008)
Object Analyst	Region-based	Commercial	PCI Geomatics
eCognition Developer	Region and edge-based	Commercial	Baatz and Shape (2000)
SPRING	Region and edge-based	Freeware	Camara et al. (1996)
EDISON	Region-based	Freeware	Comaniciu and Meer (2000)
SCRM	Region and edge-based	Freeware	Castilla et al. (2008)
RSGISLib	Region-based	Freeware	Buntin et al. (2014)
SAGA	Region and Edge-based	Freeware	Bohner et al. (2006)
Feature Analyst	Semantic	Commercial	Opitz and Blundell (2008)
ArcGIS Spatial Analyst	Region-based	Commercial	ESRI
GeoSegment	Region-based	Online tool, available upon registration	Chen (2018)

**Figure 4:**
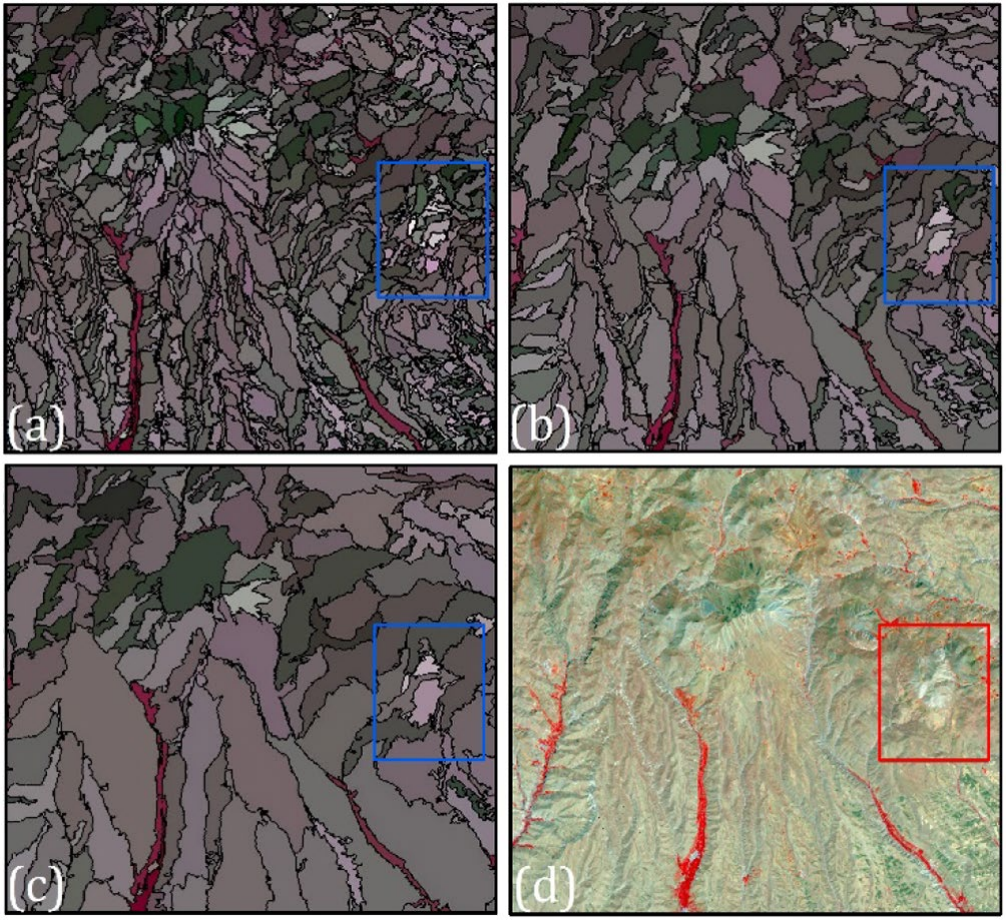
Various image segmentation scale used in MRS method: (**a**) with the scale factor of 200, (**b**) with the scale factor of 400, (**c**) with the scale factor of 600, and (**d**) original Sentinel-2 image.

Following the preparation of the image dataset and the configuration of the legend, selected bands of the image dataset and predisposing variables are used for segmentation and merging in order to split the scene into multiple components. As a result of this combined step, GEOBIA is able to produce real-world images representing real geographic objects^44^. Multi-resolution segmentation (MRS) algorithm was used in this study to generate a segmentation image using the software package eCognition Developer (Trimble Geospatial, Munich). The MRS method is a nearly complicated image and user-based algorithm, which generates a polygonal object based on the bottom-up strategy. The highly correlated adjacent pixels are initially segmented into objects. Through this process, random seed pixels are chosen that are suited appropriately for merging, and then homogeneity within the same object and heterogeneity between objects are maximized. This procedure is repeated until all the object conditions, which are controlled by color, compactness, and scale are met^45^. Of these parameters, scale is the most important factor^46^. In image segmentation, the scale parameter determines the size of the objects that appear in the image. As this parameter increases, the image becomes roughly divided. The shape parameter weights the object’s shape based on its spectral color. In this regard, spectral characteristics are more influential in segmenting images when the value is small. Compactness is the ratio between the boundary and the entire object^47^. We tested several scale parameters based on previous knowledge and used an interactive “trial-and-error” approach to segment images into homogeneous objects. We investigated different scale, shape and compactness parameters, which are presented in Table 3. Figure 4 shows the applied scale parameter to select the most appropriate scale value.

**Table 3:** Variations of segmentation parameters using the MRS method to detect and map volcanic and glacial landforms.

Landform	Scale factor	Shape factor	Compactness factor
**Volcanic landforms**			
Dacite lava	550	0.6	0.4
Caldera	150	0.8	0.2
Andesite lava	550	0.6	0.4
Volcanic cone	370	0.8	0.2
Volcanic tuff	600	0.6	0.4
**Glacial landforms**			
Glacial circus	200	0.6	0.4
Glacial valley	150	0.6	0.4
Suspended valley	290	0.8	0.2

### Object-based features extraction

**Figure 5:**
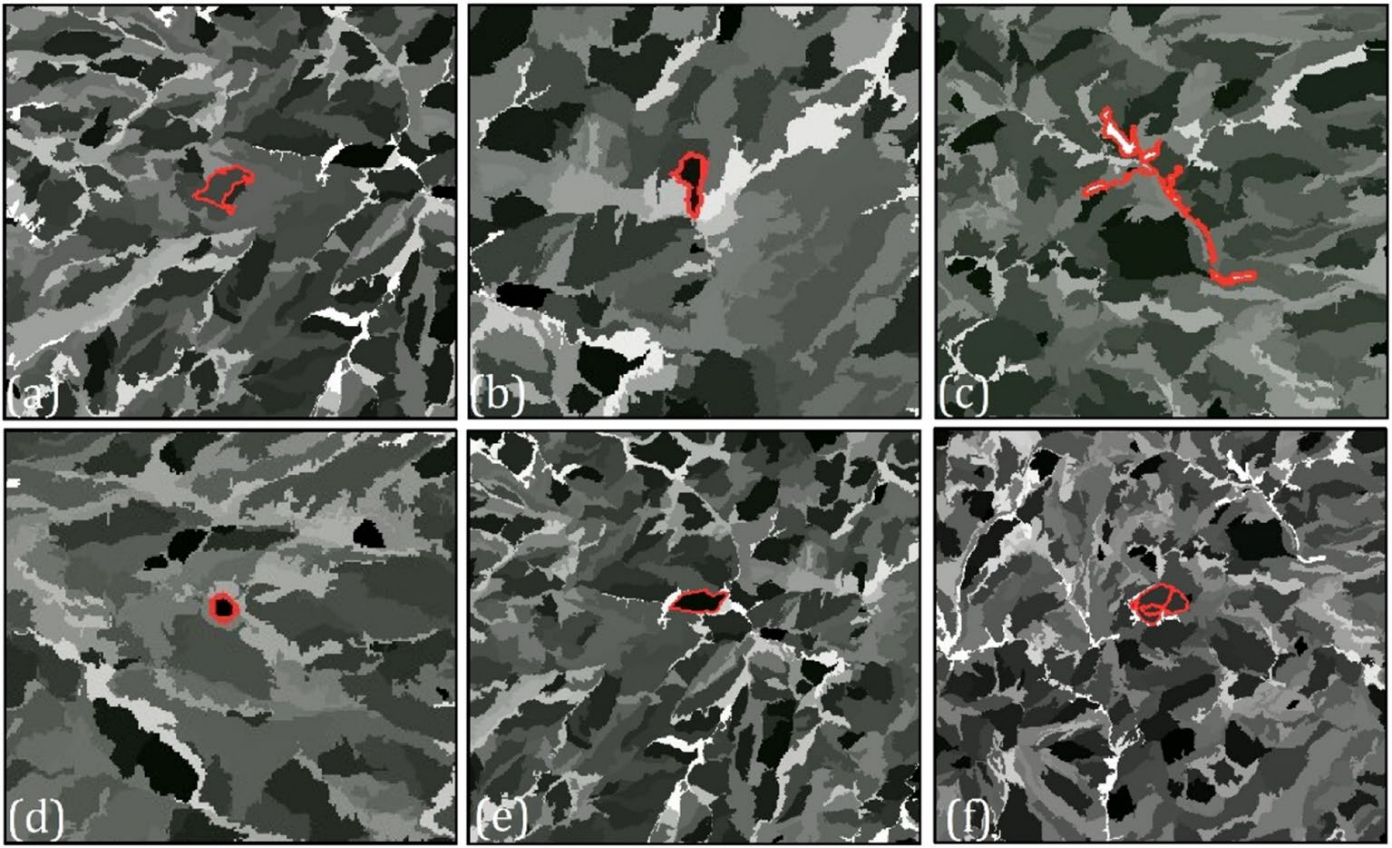
Some samples from object-based features and targeted landforms, including: (**a**) GLCM Mean (volcanic tuff), (**b**) GLCM SD (glacial cirque), (**c**) length/width (glacial valley), (**d**) shape index (caldera), (**e**) density (suspended valley), and (**f**) shape index (volcanic cone).

As shown in Table 4, the segments were selected for volcanic and glacial landforms mapping based on their spectral, textural, and geometrical characteristics. 19 variables were derived from eCognition in order to get as many variables as possible (Table 5). AND fuzzy-based operator then employed in eCognition to classify volcanic and glacial landforms based on fuzzy membership values. Not only GCPs collected by GPS, but also the existing geomorphological map, as well as aerial images, were employed to acquire training data. In sum, 935 sample points were used to identify the most appropriate threshold values for object features and to train CNN models. A rule-based approach is necessary to identify and apply object features to landform classes in GEOBIA. As a result, we incorporated training data along with the efficiency of related spatial and spectral object features for each landform class, obtained through fuzzy threshold values (Table 5). Figure 5 illustrates the performance of some of the training data over selected object-based features.

**Table 4:** Various spectral, geometrical and textural features relevant to delineating volcanic and glacial landforms and their variables.

Object-based features	Variables extracted	Properties
Spectral attributes	Brightness	Sentinel-2
	Mean	Mean Dem, Band 2, Slope, flow-accumulation, and curvature
Geometrical attributes	Standard deviation	STD curvature, and flow accumulation
	Length/width, Asymmetry, Shape index, Compactness, Elliptic fit, Density, Main direction, and Rectangular fit	
Textural attributes	GLCM entropy, GLCM contrast and GLCM STD

**Table 5:** Volcanic and glacial landforms classes and their corresponding thresholds values for object-based features.

Landform	Class	Object-based feature	Thresholds values	Fuzzy membership value
Volcanic landforms	Dacite lava	Elliptic fit	0.0.13	0.921
		Density	1.9–2.40	0.945
		GLCM contrast	9.1–9.7	0.931
	Caldera	Mean DEM	1800–3000	0.928
		Length/width	1/75–2	0.917
		Shape index	1–1/3	0.947
	Andesite lava	Main direction	130–180	0.983
		Compactness	4–25	0.919
		GLCM contrast	8.2–9	0.931
		GLCM entropy	6.7–9.2	0.899
	Volcanic cone	Mean DEM	2110–2900	0.913
		Mean band 2	400–800	0.981
		Brightness	1110–1710	0.989
		Length/width	1–1/6	0.884
		Shape index	1/6–1/9	0.963
	Volcanic tuff	Asymmetry	0.82–0.99	0.956
		Brightness	999–1060	0.982
		GLCM Mean	7.2–7.9	0.990
		Length/width	2.8–8.63	0.891
		GLCM STD	4.2–4.7	0.921
Glacial landforms	Glacial circus	Mean DEM	2500–3600	0.953
		Mean band 2	790–1030	0.918
		Mean flow-accumulation	30–450	0.956
		Mean slope	17–23	0.963
		STD curvature	5–10	0.965
		Density	2–2/5	0.908
		Shape index	1–1/5	0.914
		Length/width	1–2/5	0.987
	Glacial valley	Flow-accumulation	6000 >	1
	Suspended valley	Mean DEM	1800–2800	0.947
		Mean curvature	850–1030	0.995
		Mean slope	10°–25°	0.993
		Shape index	1–1/6	0.979
		STD Flow-accumulation	50–2000	0.947

### Convolutional neural network

**Figure 6:**
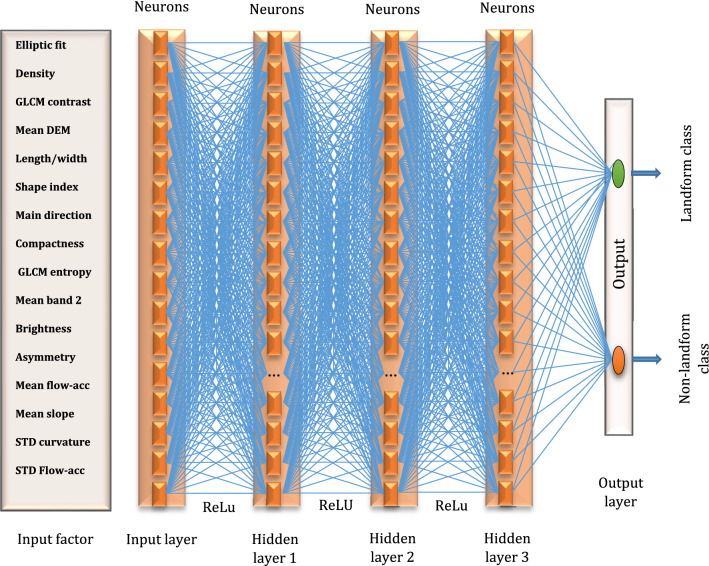
CNN structure for volcanic and glacial landforms.

A convolutional neural network (CNN) is a type of machine learning technique that works with arrays of data, such as one-dimensional signals or sequences as well as two-dimensional visible-light images or audio spectrograms^48^. Images are consisted of two-dimensional arrays of data, which makes CNN an appropriate tool for image analysis. The CNN is the most common type of deep neural network applied to remote sensing images due to its high generalization capabilities, derived from the features it extracts and its ability to train on extremely large datasets^49,50^. Neurons are the building blocks of all layers in a neural network. Each neuron represents a convolutional layer aimed at automatic feature extraction from the input image^51,52^. Figure 6 illustrates the CNN modelling structure for volcanic and glacial landforms.

### Hardware and software

This study used the Keras Python Package based on the TensorFlow backend to construct and train the GEOBIA-CNN model for volcanic and glacial landforms. The specifications of the computer system employed were Intel Core i7-6700 K, VGA (GTX 1080), HDD (256 GB SSD + 1 TB SATA) and 32 GB memory. Python programming language based on Spyder software was utilized to implement all the prediction models. In addition to user-friendliness, modularity, and extensibility, Keras provides users with easy and fast prototyping capabilities.

### Training step

We used 16 and 9 (Table 5) convolutional layers to train our CNN models for volcanic and glacial landforms, respectively (Table 6). There are several factors involved in each convolutional layer, including a pooling operation, multiple weights, and an activation function. Max-pooling was used with $2 \times 2$ filters and a two-pixel stride to down-sample the feature maps in the encoder based on a maximum operator by taking the maximum of each $4 \times 4$ matrix and putting it in the output. Landform detection in this study was done using a $128 \times 128$ pixel input window. Totally, we applied a twenty-four-layer CNN model for landform detection separately. We fed the twenty-four -layer depth CNN separately with all input window sizes of the training sample patches using nineteen variables (Table 5). In this regard, the input sample patch had $a \times a \times 19$ units, where $a \times a$ denotes the size of one layer of sample patches ($128 \times 128$), and 19 is all number of the layers required for the analysis. Several convolutions were performed on input using different filters $\left( {2 \times 2} \right)$ resulting in distinct feature maps. All these feature maps are stacked together to form the convolution layer. By stacking all features along the depth dimension, we generated the final landform outputs volume of $128 \times 128 \times 19$ by the network by using 24 different filters (one filter per convolutional layer).

**Table 6:** Characteristics of employed GEOBIA-CNN models for volcanic and glacial landforms.

Landform	Class	Activation function	Loss function	Number of convolutional layer	Optimizer
Volcanic	Dacite lava	ReLu	Cross-entropy	3	ADAM
	Caldera	ReLu	Cross-entropy	3	ADAM
	Andesite lava	ReLu	Cross-entropy	4	ADAM
	Volcanic cone	ReLu	Cross-entropy	5	ADAM
	Volcanic tuff	ReLu	Cross-entropy	5	ADAM
Glacial	Glacial circus	ReLu	Cross-entropy	8	ADAM
	Glacial valley	ReLu	Cross-entropy	4	ADAM
	Suspended valley	ReLu	Cross-entropy	5	ADAM

Layers of convolution are applied to the valid portions of the image (without any kind of padding) and they are associated with a convolution combined with an activation function to introduce nonlinearity^53^. There are different activation functions (e.g., sigmoid, softmax, tanh, hyperbolic tangent, ReLU and Leaky ReLU), which are required for the forward propagation and its derivative for backpropagation^54^. Sigmoid, tanh, hyperbolic tangent and Softmax are typically used in normal neural networks. Rectified Linear Unit (ReLu), on the other hand, is commonly used in CNN algorithms due to their superior performance^55^. Thus, ReLu function was employed in this study to train landform models. In Eq. (1), the Rectified Linear Unit (ReLU) parameters are defined as follows:

1 $$ f\left( x \right) = \left\{ {\begin{array}{*{20}c} x & {if\,x > 0} \\ 0 & {if\,x \le 0} \\ \end{array} } \right. = {\text{max}}\left( {x, 0} \right) $$

### Loss/cost function

Since training is an iterative process, the loss/cost function is necessary to quantify how good the current state of the network (with specific sets of weights) is. This function is based on the principle of increasing forecast accuracy and reducing errors in the network in order to optimize output at the lowest cost possible^56^. There are several loss/cost functions for problem-solving in classification, including Mean Squared Error (MSE), Cross-Entropy, and Mean Absolute Error (MAE), and subsequently used Cross-Entropy loss (log loss)^57^.

Since landform classification is a binary classification (0 = No Landform and 1 = Landform), Cross-Entropy is employed in this study to measure the performance of a classification model^58–60^. It is given by the following equation:

2 $$ L\left( {y, \hat{y}} \right) = - \frac{1}{N}\mathop \sum \limits_{i = 1}^{N} \left( {y_{i} \log \left( {\hat{y}_{i} } \right)} \right) + \left( {1 - y_{i} } \right){\text{log}}\left( {1 - \hat{y}_{i} } \right)) $$

where $N$ represents the number of sample datasets, $y_{i}$ denotes the actual output of ample $i$, which is equals to 0 or 1, $\hat{y}_{i}$ presents the forecasted possibility sample $i$ having output 1, and $y_{i} ,\hat{y}_{i}$ are the vectors of actual outputs and forecasted possibilities.

### Optimization

This study used ADAM to optimize the results of landform-based models. This function can replace the SGD algorithm and take advantage of AdaGrad and RMSprop, which have better performance in sparse gradients and unstable conditions, respectively^61,62^. Equations (3) and (4) are defined the ADAM optimizer:

3 $$ m_{t}^{\left( j \right)} = \beta_{1} m_{t - 1}^{\left( j \right)} + \left( {1 - \beta_{1} } \right)g_{t}^{\left( j \right)} $$

4 $$ v_{t}^{\left( j \right)} = \beta_{2} v_{t - 1}^{\left( j \right)} + \left( {1 - \beta_{2} } \right)(g_{t}^{\left( j \right)} )^{2} $$

where $\beta_{1}$ and $\beta_{2}$ are commonly chosen to be 0.9 and 0.999, respectively. The first and second moments are then bias-corrected:

5 $$ \hat{m}_{t}^{\left( j \right)} = \frac{{m_{t}^{\left( j \right)} }}{{1 - \beta_{1}^{t} }},\quad \hat{v}_{t}^{\left( j \right)} = \frac{{v_{t}^{\left( j \right)} }}{{1 - \beta_{2}^{t} }} $$

And used to weight the update:

6 $$ w_{t + 1}^{\left( j \right)} = w_{t}^{\left( j \right)} - \frac{\alpha }{{\sqrt {\hat{v}_{t}^{\left( j \right)} + \varepsilon } }} \hat{m}_{t}^{\left( j \right)} $$

where $\alpha$ is the initial learning rate, which the default value for it is 0.001.

## Accuracy assessment

### Fuzzy synthetic evaluation

GEOBIA uses fuzzy decision rules and membership values as the basis of object-based classification, which makes it a "soft classifier" approach. Due to the segmentation process, scale regulation, and fuzzy decision rules, it is difficult to assign true or false labels to objects in a binary mode^63^. As a consequence, we applied Fuzzy Synthetic Evaluation (FSE) for the accuracy assessment of the classification results. Two groups of data, including control point data and the respective rate obtained for each point are used in the FSE to calibrate the overall and per-class accuracy of classified maps using GOBIA according to two steps^64^. The first step is to compute the classification confidence or magnitude of error for each class using the Difference fuzzy function. To obtain the single accuracy value, the second step weights the Difference function categories. Following these two steps, the degree of confidence in the classification can be calculated based on the ratio of matches between sample and reference data, based on their respective interpretation confidence ratings (ICR), for which default values have been suggested^65^. A combination of GPS data, control points from Google Earth, high-resolution aerial photographs, and geomorphology maps (scale of 1/25,000) was incorporated in our research as reference datasets.

The FSE operates by assuming that $N$ is the landform classes in the classified map, labeled $C_{1}$ to $C_{N}$, organized as a set of ${\Omega } = \left\{ {C_{1} {\text{ to }}C_{N} { }} \right\}$, and that each piece sample observation is associated with one class and only one Difference category $D = \left\{ {VHCC, .., VHE} \right\}$. For the FSE appliance, let $P_{N, d}$ to be equal the proportion of observations from the map class $C_{N}$ in the Difference category $d$, and $W_{d}$ equal the assigned weight. Equation (7) can be used to calculate the estimated accuracy $P_{m}$ for map class $C_{m}$:

7 $$ P_{N} = \sum d P_{N, d} \times W_{d} { } $$

Table 7 displays Different categories of confidence in classification and magnitude of errors. The results of validation analysis using the FSE approach for volcanic and glacial landform classification are shown in Table 8. As shown in Table 8, GEOBIA demonstrates satisfactory performance for landform-based object extraction, with FSEs of 0.9204, 0.917, 0.934, 0.933, 0.921, 0.904, 0.918, and 0.909 estimated for dacite lava, caldera, andesite lava, volcanic cone, volcanic tuff, glacial circus, glacial valley, and suspended valley, respectively.

**Table 7:** The Difference function and its respective default values.

Accuracy	Level of confidence	Class map		ICR %
		Demand class	Alternative class	
Confidence in classification	Very high confidence in classification (VHCC)	*		≥ 90
	High confidence in classification (HCC)	*		≥ 85
	Acceptable confidence in classification (ACC)		*	≥ 80
	Reduced confidence in classification (RCC)	*		80 ≤
	Very reduced confidence in classification (VRCC)		*	50
Magnitude of errors	Acceptable error (AE)			50 ≤
	High error (HE)			≥ 85
	Very high error (VHE)			≥ 90

**Table 8:** Sample observation proportions for Difference categories and validation of object-based features for volcanic and glacial landforms.

Landform class	Level of confidence	Dacite lava	Caldera	Andesite lava	Volcanic cone	Volcanic tuff	Glacial circus	Glacial valley	Suspended valley
Difference categories	VHCC	0.75	0.82	0.78	0.72	0.69	0.64	0.74	0.68
	HCC	0.27	0.23	0.28	0.23	0.24	0.26	0.28	0.24
	ACC	0.3	0.03	0.04	0.03	0.07	0.11	0.02	0.08
	RCC	0.05	0.05	0.04	0.05	0.06	0.04	0.03	0.04
	VRCC	0.03	0.03	0.03	0.03	0.03	0.02	0.03	0.03
	AE	0.04	0.02	0.03	0.02	0.04	0.03	0.01	0.03
	HE	0	0	0	0	0.01	0.01	0	0.01
	CHE	0	0	0	0	0	0	0	0.00
Accuracy assessment	FSE %	0.9204	0.917	0.934	0.933	0.921	0.904	0.918	0.909

### Quantitative methods

To evaluate our CNN segmentation models for volcanic and glacial landforms, we used seven evaluation indexes, including intersection over union (IOU) values, recall (RC), precision (PC), specificity (SP), F-measure (FM), accuracy (ACC), and kappa (KP), which are defined as:

8 $$ IOU = \frac{AO \cap EO}{{AO \cup EO}} = \frac{TP}{{TP + FP + FN}} $$

9 $$ Recall = \frac{TP}{{TP + FN}} $$

10 $$ Specificity = \frac{TN}{{TN + FP}} $$

11 $$ Precision = \frac{TP}{{TP + FP}} $$

12 $$ F{\text{-}}measure = 2 \times \frac{Precision \times Recall}{{Precision + Recall}} $$

13 $$ Accuracy = \frac{TP + TN}{{TP + TN + FN + FP}} $$

14 $$ Kappa = \frac{{TP + TN - TP_{expected} - TN_{expected} }}{{TP + TN + FN + FP - TP_{expected} - TN_{expected} }} $$

15 $$ TP_{expected} = \frac{{\left( {TP + FP} \right) \times \left( {TP + FN} \right)}}{TP + TN + FN + FP} $$

$$ TN_{expected} = \frac{{\left( {TN + FN} \right) \times \left( {TN + FP} \right)}}{TP + TN + FN + FP} $$

where $AO$ is actual output; $EO$ is on behalf of expected result; $TP$, $FP$, $FN$, and $TN$ are true positive, false positive, false negative, and true negative, respectively.

Table 9 illustrates the results of CNN segmentation models for volcanic and glacial landforms. As we see in Table 9, CNN segmentation models performed well with ACC of > 0.9600 for volcanic and glacial landforms. Based on Table 9, CNN segmentation models demonstrate the highest performance of these approaches for volcanic and glacial landforms mapping, so that the ACC 0.9685, 0.9780, 0.9614, 0.9767, 0.9675, 0.9718, 0.9600, and 0.9778 were estimated for dacite lava, caldera, andesite lava, volcanic cone, volcanic tuff, glacial circus, glacial valley, and suspended valley, respectively. In addition, CNN segmentation models were performed well with KP of > 0.9000, FM of > 0.9200, SP of > 0.9700, PC of > 0.9500, RC of > 0.8600, and IOU of > 0.8500 for volcanic and glacial landforms mapping (Table 9). We also used loss and accuracy operations in Python based Spyder software to evaluate the accuracy of the integrated GEOBIA-CNN classification results for volcanic and glacial landforms mapping (Table 10). According to Table 10, CNN segmentation models achieved an accuracy of > 0.9600.

**Table 9:** Results of evaluation indexes for volcanic and glacial landforms.

Landform	Network	IOU	RC	PC	SP	FM	KP	ACC
Volcanic landforms	Dacite lava	0.8699	0.8845	0.9641	0.9885	0.9385	0.9154	0.9685
	Caldera	0.8700	0.8701	0.9581	0.9925	0.9312	0.9014	0.9780
	Andesite lava	0.8612	0.8835	0.9600	0.9847	0.9412	0.9123	0.9614
	Volcanic cone	0.8725	0.8945	0.9784	0.9899	0.9408	0.9199	0.9767
	Volcanic tuff	0.8532	0.8785	0.9671	0.9756	0.9390	0.9037	0.9675
Glacial landforms	Glacial circus	0.8513	0.8825	0.9706	0.9821	0.9249	0.9109	0.9718
	Glacial valley	0.8690	0.8758	0.9510	0.9715	0.9285	0.9149	0.9600
	Suspended valley	0.8525	0.8699	0.9607	0.9812	0.9394	0.9235	0.9778

**Table 10:** Results of loss and accuracy operations estimated using the Python based Spyder software for volcanic and glacial landforms.

Operations	Dacite lava	Caldera	Andesite lava	Volcanic cone	Volcanic tuff	Glacial circus	Glacial valley	Suspended valley
Loss	0.0045	0.0074	0.0098	0.0047	0.0124	0.0057	0.0142	0.0174
Accuracy	0.9789	0.9614	0.9689	0.9745	0.9602	0.9801	0.9678	0.9609

### K-fold cross validation

K-fold cross validation is one of the validation methods for learning-based classification approaches. The results of classification can be validated by randomly assigning the initial dataset to different groups. Here, one set is used for validation and the other K-1 set is used for training^65^. This study employed fivefold cross validation to validate the results of the landform classification. The data is divided into five sets, and one set is used for validation and the other four for training. All five sets should be processed in the same way. The results of the fivefold cross validation are given in Table 11. As we see from Table 11, the integrated GEOBIA-CNN performed well (Cross validation accuracy > 0.9400) for volcanic and glacial landforms mapping.

**Table 11:** Fivefold cross validation accuracy for volcanic and glacial landforms.

K-fold cross validation	Dacite lava	Caldera	Andesite lava	Volcanic cone	Volcanic tuff	Glacial circus	Glacial valley	Suspended valley
Fivefold	0.9632	0.9412	0.9514	0.9675	0.9499	0.9774	0.9565	0.9478

## Location and geomorphological features of the study area

**Figure 7:**
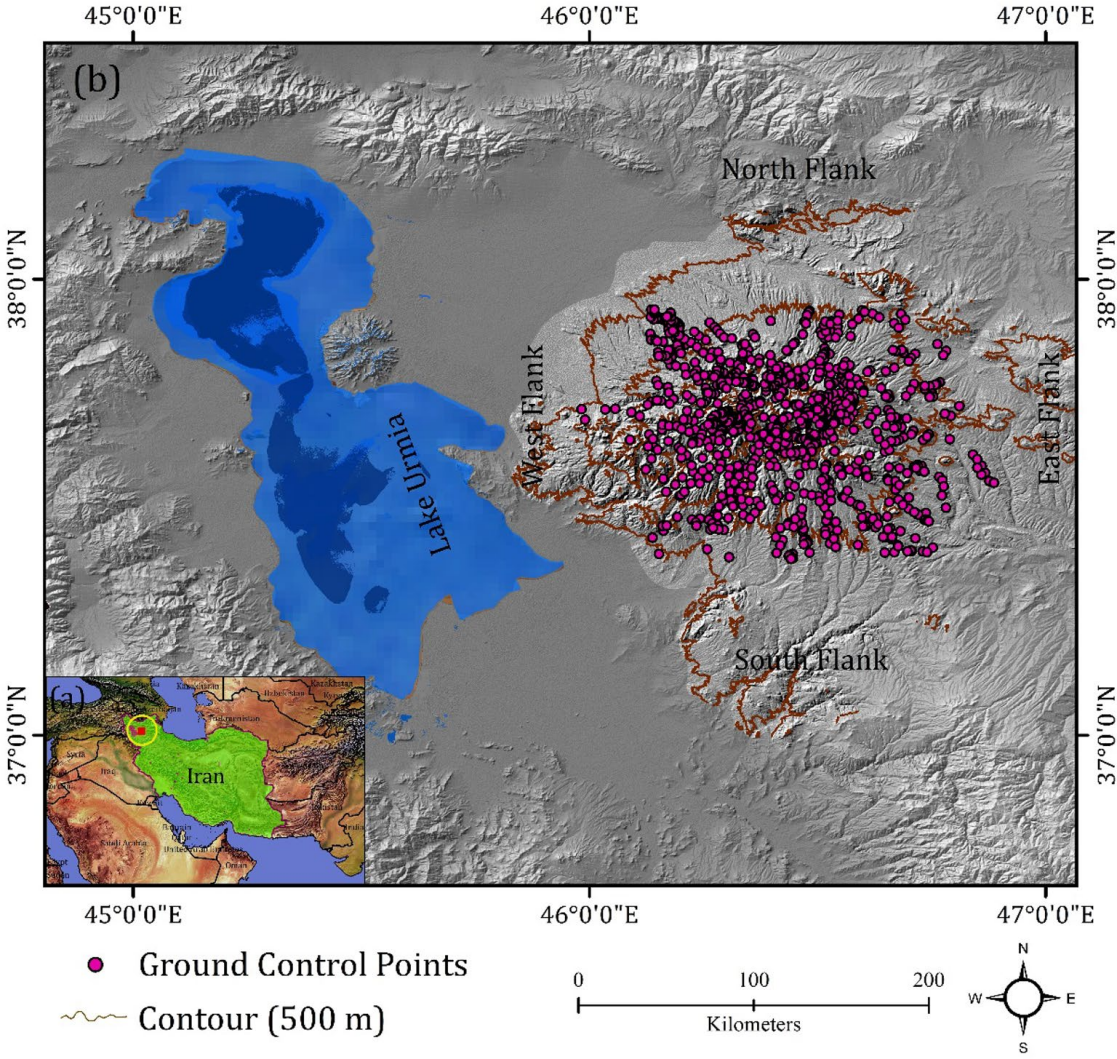
Location of study area (**a**, **b**) in the world and in Iran, and (**b**) in the north-west of Iran.

Sahand volcano is located in the north-west of Iran (Fig. 7). Geologically, Sahand volcano is very complex. The Paleocene and Miocene geologic deposits make up the majority of Sahand volcano. The Quaternary volcanic structures of Sahand volcano are aligned roughly parallel to the northwest-southeast trend of the Tabriz fault. There are 17 peaks higher than 3000 m in this volcanic complex, including Sahand's highest peak at 3707 m. Sahand's volcanic deposits cover an area of approximately 3000 km^2^, making it one of the most extensive post-collisional volcanic systems spanning eastern Anatolia, Armenia, and northwestern Iran^66^.

**Figure 8:**
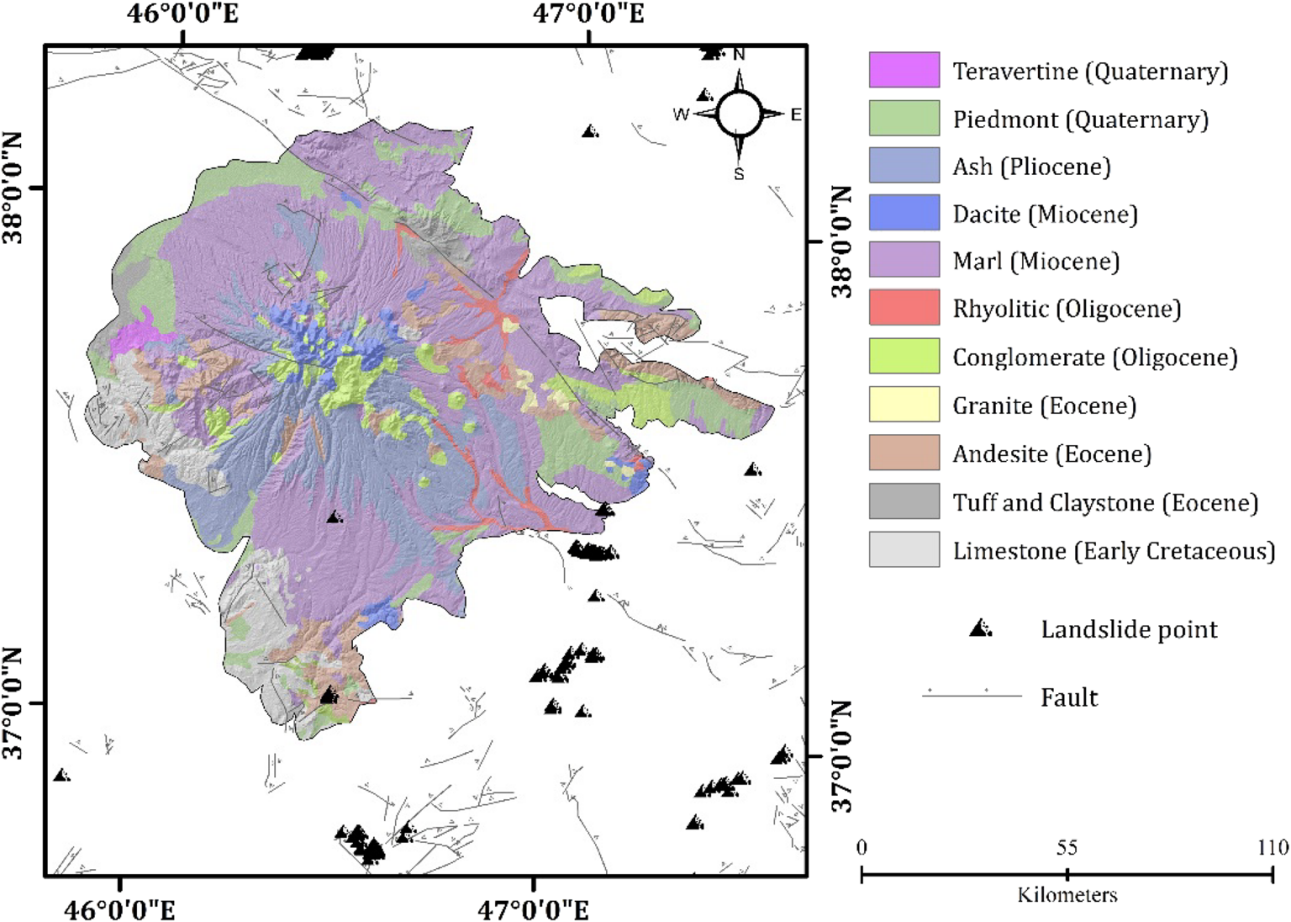
Geomorphological features of Sahand Volcano.

Researchers have discovered that during the Miocene period, lava masses were deposited on sediments through various chimneys during the Sahand eruption. As a result of the severe eruptions during this time, a large quantity of volcanic ash was dispersed over wide distances. A general study of the Sahand mass reveals three volcanic stages: a) the first occurred during the middle Miocene era and produced andesitic lavas, b) the second occurred during the late Miocene era and created the Ignimbrite that dominates all the Sahand valleys, and c) the last stage occurred during the Pliocene era and developed new volcanic cones^67^. Following the formation of Sahand, hydrological conditions and climatic conditions, as well as tectonic activity, caused a series of valleys to form and change frequently. In the Quaternary, major climatic changes played a major role in the transformation of the valleys. There is evidence of climate change in the presence of suspension valleys at the end of main valleys (the Azarshahr valley), the existence of U-shaped valleys (the Lighvan valley), the scattering of stony rocks (in the Saidabad valley), and the existence of glacial cirques (in the majority of the Sahand valleys) at the valley end^68^. Figure 8 shows the structure of Sahand volcano from a geomorphological perspective.

## Results

**Figure 9:**
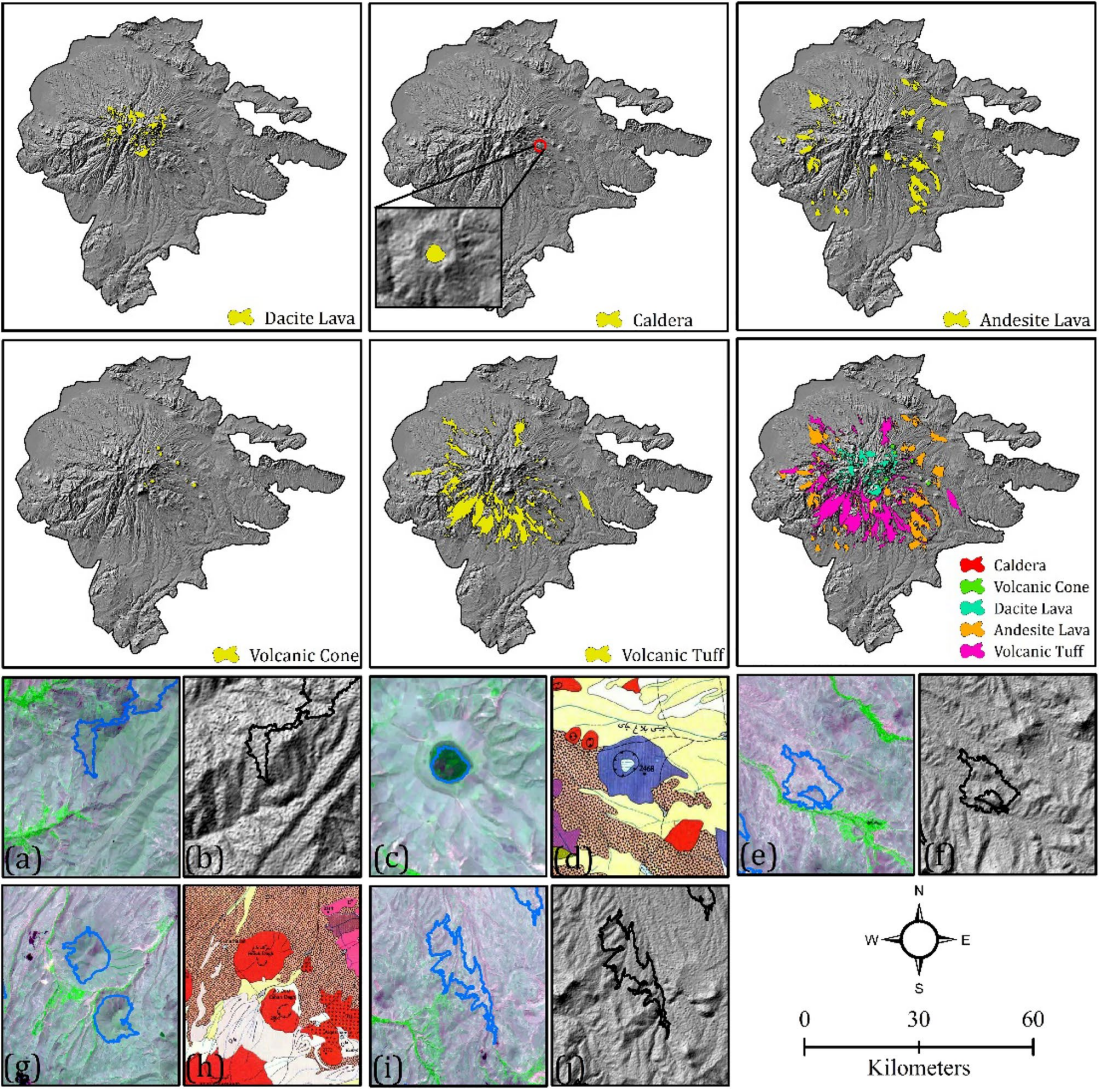
Volcanic landforms detected using the integrated approach of GEOBIA and CNN; (**a**, **b**) detected dacite lava on Sentinel-2 and curvature, respectively, (**c**, **d**) detected caldera on Sentinel-2 and geomorphological map, respectively, (**e**, **f**) detected andesite lava on Sentinel-2 and curvature, respectively, (**g**, **h**) detected volcanic cone on Sentinel-2 and geomorphological map, respectively, and (**i**, **j**) detected volcanic tuff on Sentinel-2 and curvature, respectively.

**Figure 10:**
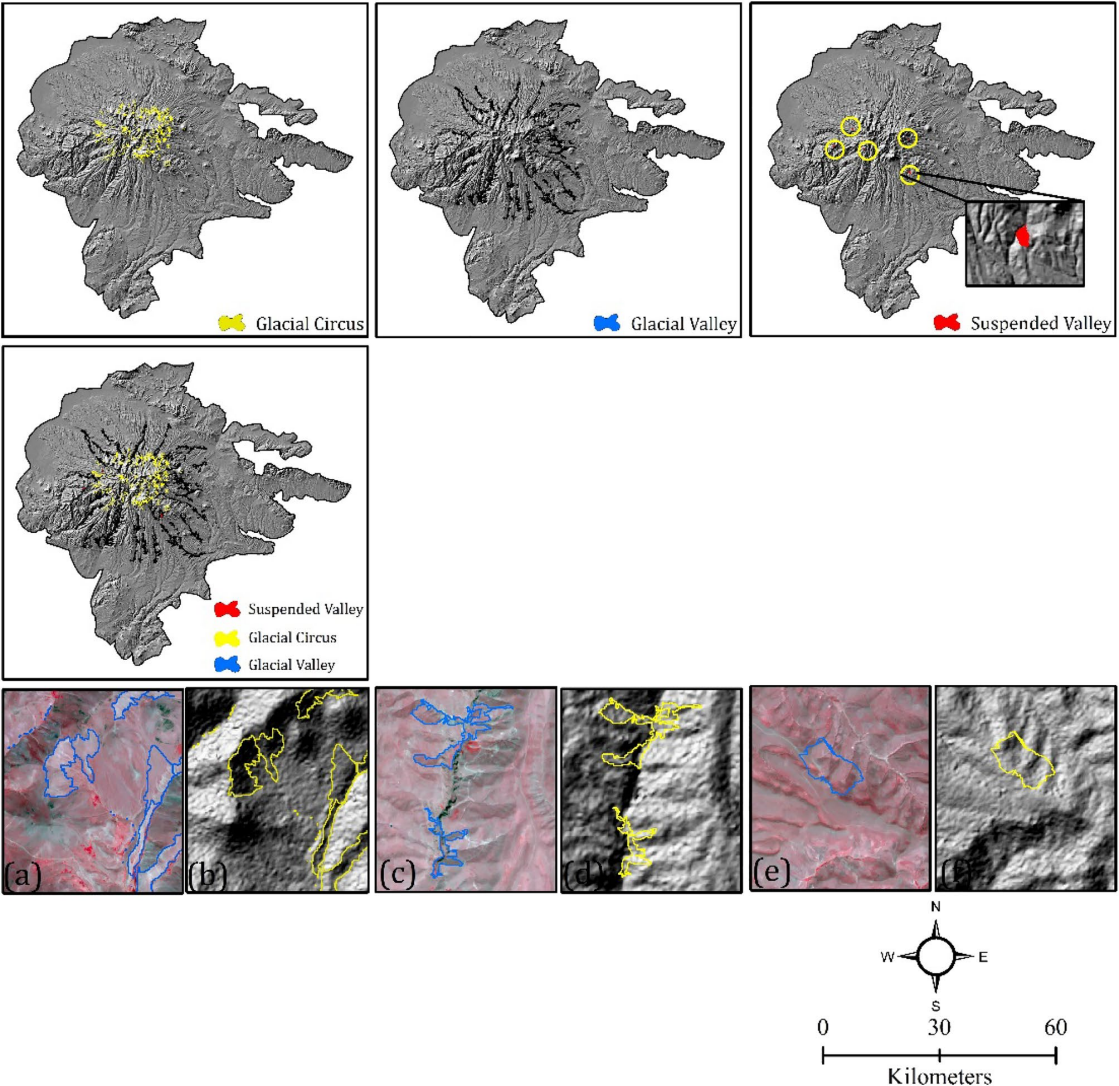
Glacial landforms detected using the integrated approach of GEOBIA and CNN; (**a**, **b**) detected glacial circus on Sentinel-2 and curvature, respectively, (**c**, **d**) detected glacial valley on Sentinel-2 and curvature, respectively, and (**e**, **f**) suspended valley on Sentinel-2 and curvature, respectively.

Figures 9 and 10 show various volcanic and glacial landforms derived from the integrated GEOBIA and CNN approach. Our first step was to segment images into meaningful objects using the MRS algorithm in eCognition software. Then, we employed geometrical, spectral, and textural features to classify volcanic and glacial landforms based on their threshold and fuzzy membership values. To construct and train CNN models, a number of 935, an inventory map as well as objects obtained from GEOBIA methods were used. 70% of the data were randomly selected as training datasets, and 30% were randomly selected as testing datasets. Our models were trained using the ReLu, Cross-Entropy and ADAM as activation, loss and optimization functions, respectively (Table 6). Integrated GEOBIA and CNN result in high performance for volcanic and glacial landforms. We see in Tables 9, 10 and 11, that GEOBIA and CNN achieved an ACC of > 0.9600 and cross validation of > 0.9400 for volcanic and glacial landforms mapping.

## Discussion

The aim of this study is to develop a method for detecting and delineating volcanic and glacial landforms automatically using GEOBIA and CNN, which allows us to develop an automatic-based methodology for detecting and mapping the Earth's landforms. Results yielded an ACC of > 0.9600 and cross validation of > 0.9400 for volcanic and glacial landforms (Tables 9, 10 and 11). Since no study has integrated GEOBIA with CNN framework for volcanic and glacial landform detection, we cannot compare our results with the literature. Our study encourages us to utilize the spectral and spatial characteristics of satellite images by integrating data from remote sensing (e.g., satellite images) with geospatial datasets (e.g., DEM, slope, aspect, and curvature). This study represents a significant step toward the development and implementation of a flexible, low-cost and automated approach for volcanic and glacial landforms detection and delineation. We introduced a unique approach for identifying and mapping a complicated and dynamic volcanic zone. We outline here a promising approach for the detection and delineation of landforms in other parts of the world. Scientists and geomorphologists can use this method to detect and delineate the earth's landforms quickly and cost-effectively.

Pixel-based DL models are limited to pixel values, whereas in this study we took advantage of several object-based advantages, including spectral, geometrical, and textural characteristics (e.g., shape index, standard deviation, GLCM, etc.). The GEOBIA approach produced satisfactory landform-based objects for training CNN models. Table 8 shows that the FSE were estimated to be 0.9204, 0.917, 0.934, 0.933, 0.921, 0.904, 0.918, and 0.909 for dacite lava, caldera, andesite lava, volcanic cone, volcanic tuff, glacial circus, glacial valley, and suspended valley, respectively. The pixel-based context ignored spatial properties, so each pixel of surrounding objects had the same spectral behavior as a landform. Nevertheless, GEOBIA uses geometrical properties such as length and width to filter glacial landforms such as glacial valleys quickly, improving precision values. In the complex mapping of objects such as landforms, even complex algorithms, such as DL models applied to pixels, are limited. The use of GEOBIA allows to mitigate some of the limitations of DL in detecting landforms by taking advantage of knowledge-based rule-sets.

Our study indicates that the GEOBIA approach is effective at extracting features, which allows us to obtain an accurate classification result during model training. As part of an object-based landform detection process, GEOBIA allows us to make use of objects' features, spatial relationships, and expert knowledge during the segmentation, classification, and validation processes. We found that integrating spatial features (e.g., shape index, width/length) with spectral information (e.g., brightness) enabled us to efficiently detect landform-derived objects. Satellite imagery generally shows high brightness values for tuff formations and zones as well as dacite lava due to the exposure of fresh rock outcrops (Table 5). Therefore, we can outline these geological formations efficiently by applying spectral features (e.g., brightness). Additionally, results indicated that desiccated riverbeds and sandy roads might also exhibit higher brightness values due to similar spectral reflectance characteristics. Through the incorporation of geometric parameters (other than spectral values), GEOBIA allows for the discrimination and delineation of tuff formations. Based on the shapes of landforms, such as a circle (Volcanic Cone and Caldera) or linear shape (Glacial Valley), geometric features such as shape index, density and compactness are useful for identifying and detecting them. As far as textural features are concerned, the grey level co-occurrence matrix (GLCM) based on contrast and standard deviation was identified as being the most efficient for volcanic and glacial landform-based objects. Thus, we evaluated the feasibility of a landform class based on the GLCM analysis of sub-objects which helps to evaluate highly textured data. Using spectral features of object images together with information derived from GIS spatial analysis, such as DEM and its derivatives, such as flow accumulation, slope, curvature, and aspect, can be efficiently applied to detect volcanic and glacial landforms. A high level of confidence was also gained for volcanic and glacial landform outlining using spatial and geometric features, including length/width, shape index, and roughness (Table 5).

Comparing CNN with traditional machine learning algorithms (e.g., SVMs, decision trees), we find that it produces better results in identifying landforms^17–19^. However, SVMs had higher specificity in most cases, up to 10% in comparison with CNNs. SVMs with higher specificities are more effective at detecting negative cases; that is, landforms that don't exist. It is, however, not as accurate as CNN when it comes to detecting landforms. As a result of this higher specificity, for most examples, SVM missed volcanic cones in the image. Additionally, the SVM approach is only capable of detecting radially symmetric landforms, since the model feature extractor that we chose is optimized for rotationally invariant landforms rather than linear ones. CNN, on the other hand, calculates its own input features based on the training datasets, so they can be readily adapted to a wide range of classification tasks. Finally, the computation complexity for traditional machine learning algorithms, such as SVMs, increases with the number of samples. Therefore, they have a worse generalization ability than deep neural networks, which can fit large amounts of data and perform well on unseen data.

On average, our GEOBIA-CNN approach managed to map volcanic and glacial landforms with an ACC of > 0.9600 and cross validation of > 0.9400. The IOU ranges from 0.8500 to 0.8700, RC from 0.8600 to 0.8900, PC from 0.9500 to 0.9700, SP from 0.9700 to 0.9900, FM from 0.9200 to 0.9400, KP from 0.9000 to 0.9200, and ACC from 0.9600 to 0.9700 indicating the highest performance of the integrated approach. Maps derived from GEOBIA could be used as auxiliary data for volcanic and glacial inventories. With our GEOBIA-CNN method, manual inventories could be reduced in uncertainty due to individual inconsistencies and subjectivity. Using our method, we could provide a landform outline base product that could be manually refined to create a finalized volcanic and glacial inventory map, thereby reducing the amount of manual digitization required. Using GEOBIA and CNN in combination has several key advantages over traditional approaches. The first aspect is that inactive glacial valleys can be identified, which would not be possible using conventional techniques alone since they are essentially non-existent. In spite of this, these features remain important for water resources and hydrology in the region. Furthermore, we employ Sentinel-2 imagery with 10 m resolution instead of very high resolution and expensive satellite imagery. The integrated approach of GEOBIA and CNN can also be applied in volcanically active regions, providing a solution to the geomorphologists’ problem in regions like volcanoes.

There are, however, some limitations to our method. It is critical to have sufficient and reliable inventory data for a given region in order to train the CNN model. While it is not necessary to have spatially complete training data, it can still be challenging for completely unstudied volcanic regions. Second, due to the CNN's reliance on identifying recurring spectral patterns and textures, it can misclassify volcanic tuff as a glacial circus, which shares the same spectral and textural characteristics. By using GEOBIA reshaping, however, false positives are mitigated to an extent that cannot be achieved with CNN alone. Objects with irregular shapes, spectral properties, or morphological properties can be excluded using GEOBIA. In this way, GCPs can assist analysts in identifying landforms manually; however, by running the entire process through GEOBIA, individual landform polygons can be obtained automatically. Based on the results of this research, our future research will focus on applying different DL algorithms such as deep multilayer perceptron and AlexNet and comparing their efficiency when integrating them with GEOBIA-CNN.

One of the major advantages of the proposed method is that it provides a reproducible, directly transferrable method to map volcanic and glacial landforms in the dynamic and difficult conditions of volcanoes based on several predisposing variables (e.g., DEM, slope, aspect, curvature, flow accumulation, and satellite imagery). The results are easily implemented in a GIS mapping environment, and can therefore be easily edited and incorporated into a general mapping procedure along with other landforms. The proposed approach outlined here is also of interest to the wider scientific community, for both planetary and submarine volcano research. Digital elevation models are a primary data source for mapping and investigating submarine volcanoes, for determining both their distribution, landforms and erupted volumes.

## Conclusion

Based on the results of this study, an integrated approach of GEOBIA and CNN provides a new method for understanding geomorphological units, especially volcanic and glacial landforms, which have been largely overlooked. Due to the limited prior research exploring the efficiency of the integrated approach of GEOBIA and CNN, identifying and developing the most robust landform modeling technique is still a serious challenge. The level of classification accuracy (> 0.96.00) estimated using integrated GEOBIA and CNN suggests that this technique can be satisfactorily applied for volcanic and glacial landform detection and delineation. A landform model based on GEOBIA and CNN becomes more efficient when combined with GIS-based data and remote sensing datasets. Our results indicate that the proposed technique can achieve the satisfying accuracy of a classification and can increase the accuracy of existing geomorphological maps.

It is increasingly critical to develop new methods and algorithms for data-driven approaches as quickly and efficiently as possible, as remote sensing and a range of earth observation products (e.g., high-resolution satellite imagery) continue to advance. Our proposed method can be widely employed to increase the accuracy of existing geomorphological as well as geological maps. The results of this study would help relevant researchers in geomorphology, geography as well as geology to understand the mechanism of landform evolution. The model introduced here can be used in similar areas with volcanic and glacial landforms. The results of the present study demonstrate that the combined model outperforms the other models in terms of considering various predisposing factors for landform mapping. The ease of use and high level of accuracy of the introduced model make it a valuable tool for future landform mapping.

## Data Availability

The datasets that support the findings of this study are available from the corresponding author on reasonable request.
